# Clinical PET Imaging of Microglial Activation: Implications for Microglial Therapeutics in Alzheimer’s Disease

**DOI:** 10.3389/fnagi.2018.00314

**Published:** 2018-10-08

**Authors:** Zhiwei Shen, Xinjie Bao, Renzhi Wang

**Affiliations:** Department of Neurosurgery, Peking Union Medical College Hospital, Peking Union Medical College, Chinese Academy of Medical Sciences, Beijing, China

**Keywords:** microglia, therapy, Alzheimer’s disease, imaging, phenotype

## Abstract

In addition to extracellular β-amyloid plaques and intracellular neurofibrillary tangles, neuroinflammation has been identified as a key pathological characteristic of Alzheimer’s disease (AD). Once activated, neuroinflammatory cells called microglia acquire different activation phenotypes. At the early stage of AD, activated microglia are mainly dominated by the neuroprotective and anti-inflammatory M2 phenotype. Conversely, in the later stage of AD, the excessive activation of microglia is considered detrimental and pro-inflammatory, turning into the M1 phenotype. Therapeutic strategies targeting the modulation of microglia may regulate their specific phenotype. Fortunately, with the rapid development of *in vivo* imaging methodologies, visualization of microglial activation has been well-explored. In this review, we summarize the critical role of activated microglia during the pathogenesis of AD and current studies concerning imaging of microglial activation in AD patients. We explore the possibilities for identifying activated microglial phenotypes with imaging techniques and highlight promising therapies that regulate the microglial phenotype in AD mice.

## Introduction

Alzheimer’s disease (AD), the most common cause of dementia, was first described by Dr. Alois Alzheimer in 1907 (Vishal et al., 2011). From decades of research, it is thought that AD is caused by neuronal and synaptic loss following deposition of extracellular β-amyloid (Aβ) plaques and intracellular neurofibrillary tangles (NFTs) (Li et al., 2016). Paradoxically, none of the therapeutic approaches targeting Aβ or NFTs have yet achieved satisfactory outcomes in AD patients. In recent years, an increasing number of studies have recognized that neuroinflammation, mainly driven by activated microglia, also contributes to AD pathogenesis (Calsolaro and Edison, 2016; Ransohoff, 2016a; Bolos et al., 2017; Mathys et al., 2017; Venegas et al., 2017). Activated microglia, just like macrophages in peripheral tissues, largely exist in two polarized states, namely the M1 phenotype and the M2 phenotype (Wes et al., 2016; Subramaniam and Federoff, 2017; Xu et al., 2017). Although, it has been realized that a simple M1 or M2 phenotype may not capture the whole status of activated microglia (Ransohoff, 2016b; Tang and Le, 2016; Plescher et al., 2018), this classification serves as a crucial guide for microglia-targeted treatment in AD patients. Commonly, the M1 phenotype is associated with the release of pro-inflammatory cytokines, such as tumor necrosis factor (TNF)-α and interleukin (IL)-1β, whereas the M2 phenotype is accompanied by the production of anti-inflammatory molecules, such as IL-10 and IL-4 (Jimenez et al., 2008; Malm et al., 2015). In light of the opposite states of microglial activation, microglia-targeted therapies should be aimed at changing the microglial status.

However, status modifications of microglial cells are very complex. Microglial activation is accompanied by many changes, including morphology, secretory profile and proliferative response (Streit et al., 2014), so altering the cell phenotype involves diverse aspects of microglia. In addition, cumulative evidence indicates that the neuroprotective M2 microglia gather at the preclinical stage of AD, whereas the neurotoxic M1 microglia peak at the clinical stage (Jimenez et al., 2008; Sarlus and Heneka, 2017). Total microglia activation inhibition will inevitably impair the beneficial function of M2 microglia, thus this kind of treatment is inappropriate and inadvisable. Immunotherapy that boosts or tempers inflammation should be dependent on the microglial phenotypes during the specific stages of AD in patients.

Because of the various survival times of microglia and the complex stimuli in the microenvironment of the AD brain, concurrent existence of both the M1 and M2 phenotypes *in vivo* is common, and understanding which phenotype is dominant at each phase is necessary to take appropriate therapeutic measures. However, accurate biomarkers of the *in vivo* microglial phenotype have not been discovered yet, and so it is difficult to identify their exact subtypes. Thanks to the rapid development of imaging technologies, imaging microglial activation *in vivo* is already possible. Although this kind of technology is unable to discriminate the exact phenotype of activated microglia, we can distinguish the phenotype according to the disease stage. Thus, this technique might be applied to direct the treatment of AD patients in the hospital. Regardless of whether the result is M1 or M2, an anti-microglia agent targeting the M1 phenotype or a pro-microglia agent targeting the M2 phenotype would be most beneficial for AD patients. In this review, we summarize the critical role of microglial activation in the pathogenesis of AD, review the published studies on imaging of microglial activation, and highlight a novel therapeutic approach that exerts neuroprotective effects by modulating microglial activation states in AD patients.

## Pathological Significance of Microglial Phenotypes in AD

**FIGURE 1:**
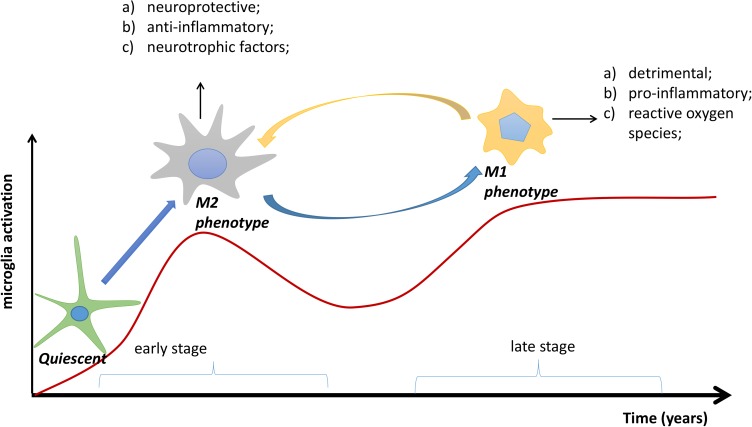
The transformation process of microglial phenotypes in Alzheimer’s disease (AD). In the early stage of AD, quiescent microglias are activated to the M2 phenotype by accumulating Aβ plaques; in the later stage, the M2 phenotype may be transformed to the M1 phenotype. Overall, the M2 phenotype is neuroprotective and anti-inflammatory, whereas the M1 phenotype is detrimental and pro-inflammatory. Red line: degree of microglial activation.

Microglia are the resident macrophages in the central nervous system (CNS), accounting for 10–15% of all cells in the brain (Lawson et al., 1992). Normally, they exist in a quiescent state, constantly monitoring their microenvironment, but can be activated by surrounding stimuli. Amyloid plaques, the pathological hallmark of AD, are able to attract and stimulate microglia *in vivo* (Jung et al., 2015; Chen et al., 2016; Yin et al., 2017). This activation process is diverse and includes microglial proliferation, increased secretion of inflammatory factors, cell surface receptor expression, and a morphological change from ramified to amoeboid (Malm et al., 2015; Wolf et al., 2017). In response to growing Aβ plaques, activated microglia can acquire different phenotypes that play dual roles during AD pathogenesis. The early activation of microglia that attempt to clear Aβ is considered as protective and anti-inflammatory (Jimenez et al., 2008; Shen et al., 2017), equivalent to the M2 phenotype. Typically, M2-polarized microglia show enhanced phagocytosis (Mandrekar-Colucci et al., 2012), upregulated expression of Ym1 and arginase 1 (ARG1) (Zhang et al., 2017), and increased secretion of anti-inflammatory cytokines, such as IL-4, IL-10, IL-13, and transforming growth factor-β (Butovsky et al., 2005; Zhou et al., 2012). However, with continuous development of AD pathology, microglia with an M2 phenotype may become dysfunctional over time and be replaced by cells with an M1 phenotype, which is detrimental and pro-inflammatory (Bronzuoli et al., 2016; **Figure 1**). Accordingly, M1-polarized microglia are associated with relatively poor phagocytosis and increased secretion of pro-inflammatory cytokines, such as IL-1, IL-6, IL-12, IL-18, and TNF-α (Malm et al., 2015). In support of this idea, one study has demonstrated that increased cortical and hippocampal neurodegeneration induces a shift of activated microglia from M2 to M1 (Kumar et al., 2016). Moreover, M2 microglia in amyloid precursor protein and presenilin 1 (*APP/PS1*) mutant mice at 6 months of age could, with ongoing Aβ accumulation, switch to an M1 microglial phenotype at 18 months of age (Jimenez et al., 2008).

The pro-inflammatory factors released by M1 microglia have many detrimental effects, resulting in the exacerbation of AD progression. For example, IL-1, a promotor of IL-6 production and microglial activation, is upregulated in AD mice and believed to promote Aβ deposition (Heneka and O’Banion, 2007). Though a study has shown that IL-1β overexpression decreases plaque area and frequency (Shaftel et al., 2007), it should be noted that this study was performed in *APP/PS1* mice only 6 months of age, which is regarded as an early stage of AD progression. IL-6 has recently been found to be a useful biological marker that significantly correlates with the severity of cognitive impairment (Lai et al., 2017). Moreover, some studies have suggested IL-6 is intimately associated with amyloid precursor protein (APP) metabolism (Kalman et al., 1997; Leblhuber et al., 1998; Cojocaru et al., 2011). TNF-α, a dual factor relaying both neuron death and neuroprotection, has been shown to be involved in AD-related neuroinflammation and amyloidogenesis via β-secretase (Cheng et al., 2014). Further, soluble TNF receptors promote the conversion from mild cognitive impairment (MCI) to dementia in patients with MCI (Buchhave et al., 2010; Diniz et al., 2010) and stimulate Aβ production through activation of transcription factors (Buchhave et al., 2010). In addition, increased levels of pro-inflammatory cytokines including IL-1, IL-6, and TNF inhibit the phagocytosis of Aβ in the brain of AD model mice (Stamouli and Politis, 2016).

Conversely, M2 microglia provide neuroprotective effects in many ways. Firstly, the anti-inflammatory cytokines they secrete, such as IL-4, IL-10, and TGF-β, suppress pro-inflammatory cytokine production and action (Rubio-Perez and Morillas-Ruiz, 2012). In addition, IL-4 inhibits IFN-γ and decreases the expression of TNF-α and nitric oxide (Chao et al., 1993). Other studies have shown that IL-10 mediates enhanced neurogenesis (Kiyota et al., 2012) and maintains neuronal homeostasis by inhibiting apoptotic pathways (Strle et al., 2001). In addition, ARG1 and Ym1, the typical biomarkers of M2 microglia, can repair damage to the extracellular matrix (Varnum and Ikezu, 2012; Tang and Le, 2016). Further, the secretion of Aβ-degrading enzymes, like insulin-degrading enzyme (Kawahara et al., 2014), is enhanced, along with phagocytic activity (Mandrekar-Colucci et al., 2012). In contrast, a few studies have revealed that anti-inflammatory signaling might be detrimental in AD (Town et al., 2008; Chakrabarty et al., 2012, 2015; Guillot-Sestier et al., 2015). The underlying mechanism of this effect has not been explained or demonstrated with solid support by the authors. Thus, further studies about the unwanted side-effects of anti-inflammatory cytokines are still required for AD.

Apart from the M1 and M2 phenotypes, some new microglial activation phenotypes have been recently identified. For example, disease-associated microglia (DAM), a newly identified subset of microglia, have been demonstrated to have a unique transcriptional and functional signature (Keren-Shaul et al., 2017). In the study, the authors characterized the involvement of microglia in a mouse model of AD by using single-cell sorting, revealing that DAM are activated through a two-step process; that is, from homeostatic microglia to an intermediate state and finally to a fully activated DAM state. The first step is *TREM2*-independent, whereas the second step is *TREM2*-dependent. DAM are associated with sensory mechanisms, phagocytosis and lipid metabolism, so Keren-Shaul et al. (2017) suggest that the microglia have the potential to restrict AD progression. However, it remains contentious whether microglia’s role is protective or injurious (Brown and St George-Hyslop, 2017). Further study needs to test whether DAM depletion affects disease progression in AD and whether excessive microglial phagocytosis is deleterious. In addition, using ultrastructural analysis, another study has identified a new microglial phenotype, named dark microglia, that also plays a significant role in the pathological remodeling of neuronal circuits in *APP/PS1* mice (Bisht et al., 2016). These phenotypes will need to be better understood for the development of new microglia-targeted treatments.

## The Therapeutic Effects of Drugs That Act on Microglia in AD

Despite decades of study, currently there are still no effective therapies available for AD patients, and this mainly arises from incorrect treatment targets. Past studies have focused extensively on reducing Aβ or tau levels. In recent years, research on microglia as potential targets for AD treatment have gained attention, especially strengthened by a recent discovery in a genome-wide association study (Sims et al., 2017), which found that microglia with variants in PLCG2, ABI3 and TREM2 genes contribute directly to the development of AD. Similarly, a review elucidated that several genes expressed in microglia, including APOE, TREM2, CD33, GRN and IL1RAP, alter AD risk, and therefore could be considered as microglial therapeutic targets (Wes et al., 2016). In addition, microglial functions are largely affected by aging. In the aged brain, microglia are chronically impaired and dysfunctional, transforming into an M1 phenotype, as evidenced by reduced process motility, aberrant morphology, and decreased phagocytic ability (Miller and Streit, 2007; Damani et al., 2011; Daria et al., 2017). However, such microglial dysfunction might be reversible (Daria et al., 2017), and the restored microglia may be able to remodel the disease pathology in several ways, including phagocytosis of Aβ plaques, secretion of Aβ-degrading enzymes and neurotrophic factors, inhibition of the release of pro-inflammatory factors, and promotion of endogenous neurogenesis (Serini and Calviello, 2016; Shen et al., 2017). Moreover, M2 microglia have the ability to promote remyelination in the CNS (Miron et al., 2013).

Therefore, therapies should reflect the disease stage of a patient and be more specific, such as enhancing the M2 phenotype in the early stage by suppressing activation of M1 or boosting the switch from M1 to M2. Firstly, as stated above, microglia predominantly adopt the M2 phenotype in the early stage, which is beneficial and anti-inflammatory. Thus, early interventions aimed at suppressing overall microglial activation are destructive and should be avoided. Rather, treatment options that increase M2 microglia could promote a neuroprotective effect and contribute to the survival of neurons. For example, a study by Jin et al. (2017) has reported that peritoneal dialysis can enhance the Aβ phagocytosis function of microglia in *APP/PS1* mice and reduce amyloid-beta plasma levels in humans. Secondly, M1 cells are typically detrimental. They can produce a lot of pro-inflammatory factors like nitric oxide, IL-1, IL-6, and TNF (Koning et al., 2009). M1 cells predominate in the later stage of AD and greatly aggravate disease progression. Therefore, treatments that interfere with the activation of M1 may be effective in halting lesion development. An increasing number of studies have shown that regulating the ratio of M1/M2 or switching the detrimental M1 phenotype toward the beneficial M2 phenotype can produce a good clinical outcome (Biagioli et al., 2017; Chen et al., 2017; Yao et al., 2017). We recommend that such treatment should be started as early as possible and would be best during the time right when M2 microglias are transitioning to M1 microglia. To sum up, these therapeutic approaches focus on not only decreasing the harmful effects but also enhancing the beneficial effects of neuroinflammation by modulating the ratio of M1/M2. Such microglia-targeted therapies are potential avenues for AD treatment.

## Current Imaging of Microglia *In Vivo* in AD Patients

Undoubtedly, activation of microglia plays a significant role in the underlying pathology of AD. Because of dynamic and context-dependent properties, microglial phenotypes are hard to identify. Understanding which phenotype dominates *in vivo* at specific times will definitely help in the selection and implementation of efficacious therapeutics. Imaging microglial activation *in vivo* is a new research field that is providing a better understanding of their function over time. Many imaging techniques have been studied for microglial visualization, such as confocal microscopy, multiphoton microscopic imaging, magnetic resonance imaging (MRI), and Positron emission tomography (PET) (Venneti et al., 2009). The detailed analyses of each technique have been well-reviewed by Venneti et al. (2009). In the following paragraphs, we mainly aim to review the possibility of imaging microglial activation in AD patients using MRI and PET, which have the potential for further *in vivo* application in human beings.

High-field MRI scanners, particularly 7 T MRI, have been increasingly used as powerful tools for AD studies *in vivo*. The main neuroimaging biomarkers of 7 T MRI that have been identified for AD patients are neuroanatomical atrophy, molecular characterization of hypo-intensities and micro-infarcts (Ali et al., 2015). Recent studies have shown colocalization of ferritin with microglia in AD patient samples (van Duijn et al., 2013; Kwiatek-Majkusiak et al., 2015), and high-field MRI is quite sensitive to microscopic iron. Thus, it might be possible to detect microglial activation by ultra-high resolution MRI. For instance, a study by Zeineh et al. (2015) has demonstrated that there are numerous small MR hypo-intensities within the hippocampus in late-stage AD specimens but not in controls, indicating small hypo-intensities are imaging features of activated iron-containing microglia. Interestingly, another study that assessed metabolite levels using magnetic resonance spectroscopy found that macromolecule and lipid levels (ML9) might represent a biomarker related to the microglial phenotype in mice (Pardon et al., 2016), which thus might help distinguish microglial activation in AD mouse models. Both the above-mentioned studies have successfully explored the visualization of microglial activation with high-field MRI in mouse or human brain specimens, laying the foundation for future *in vivo* application in human beings.

Compared with MRI, imaging of microglial activation using PET is much more practical and has been greatly studied in AD patients. **Table 1** provides a list of most of the studies that have been published in the PUBMED database with the key words of “microglia,” “phenotype,” “PET,” and “Alzheimer’s disease.” The 18 kDa translocator protein (TSPO) is the main target for PET studies, and is mainly expressed by activated microglia (Cosenza-Nashat et al., 2009). Though it has been reported that TSPO is expressed both in brain microglia and blood immune cells (Kanegawa et al., 2016), a study by Narayan et al. (2017) has demonstrated that TSPO is downregulated in blood macrophages. In addition, another study has reported the presence of monocyte-derived macrophage infiltration in a very aged mouse model of AD (Martin et al., 2017). There is evidence that TSPO radioligand binding correlates with the abundance of activated microglia (Chen and Guilarte, 2008; Venneti et al., 2008). To date, many kinds of TSPO PET tracers have been used; [11C]-PK11195 is representative of the first generation of PET tracers and has been most widely used. Nevertheless, [11C]-PK11195 has several limitations, including low brain uptake, low bioavailability and high lipophilicity (Ching et al., 2012). In comparison, the second generation of TSPO ligands, such as [11C]-PBR28 (Kreisl et al., 2010), [18F]-DPA-714 (Golla et al., 2015) and [11C]-DPA-713 (Endres et al., 2009), exhibit higher affinity and better kinetic characteristics. However, they are sensitive to a polymorphism of the TSPO gene (Ala147Thr) (Owen et al., 2012). The genotypic status of the patients could be classified into low, high, and mixed affinity binders based on this polymorphism (Lagarde et al., 2018). In **Table 1**, there are three clinical studies that have used the second generation of TSPO ligands that require genetic information, including [11C]-PBR28 and [18F]-DPA-714. Among them, the studies with TSPO polymorphism genotyping have excluded the individuals with low affinity binders (Kreisl et al., 2013; Hamelin et al., 2016), so their results are more valuable than those that lack TSPO genotype information (Golla et al., 2015).

**Table 1:** Clinical studies of microglial activation by PET imaging in AD patients.

Subjects	Age	Tracer(s)	Binding change (PET)	Reference
8AD; 15control; 1MCI	AD:	[11C] (R)PK11195	Increase in MCI and AD	Cagnin et al., 2001
	66.1 ± 5.3 years			
	MCI:			
	75 years			
22AD; 14MCI	MCI:	[11C] (R)PK11195 and [11C]-PIB	Increase in MCI and AD	Okello et al., 2009
	66.6 ± 9.6 years			
	AD:			
	64.9 ± 6.4 years			
10AD; 6control	AD:	[18F]-DPA-714; No TSPO genotyping	Not significant between AD and control	Golla et al., 2015
	71.8 ± 9.9 years			
	Control:			
	64.5 ± 5.5 years			
26AD; 38 MCI;	MCI:	[18F]-DPA-714 and [11C]-PIB; With TSPO genotyping	Increase in both MCI and AD	Hamelin et al., 2016
	67.8 ± 9.1 years			
	AD:			
	68.3 ± 12.1 years			
7AD; 8MCI	MCI:	[11C]-DED	Increase in MCI and AD; most in MCI	Carter et al., 2012
	62.6 ± 7.5 years			
	AD:			
	65.1 ± 6.3 years			
10AD; 7MCI; 10control	MCI:	[11C]-DAA1106	Increase in MCI and AD	Yasuno et al., 2012
	67.1 ± 9.9 years			
	AD:			
	70.2 ± 7.4 years			
19AD; 10MCI	AD:	[11C]-PBR28; with partly TSPO genotyping	Increase in AD; early-onset > late-onset; age-dependent	Kreisl et al., 2013
	63.1 ± 8.8 years			
	MCI:			
	72.6 ± 9.7 years			
19AD; 10MCI; 21control	AD:	[11C] (R)PK11195	Subtle increase in AD; no in prodromal	Schuitemaker et al., 2013
	69 ± 8 years			
	MCI:			
	72 ± 6 years			
8AD; 14control	AD:	[11C] (R)PK11195	Increase in AD; age-dependent	Fan et al., 2015
	66 ± 4.8 years			
	Control:			
	65 ± 5.5 years			
8AD; 17MCI	MCI:	[11C]-DED	Increase in AD and MCI; higher in MCI; decline with age	Rodriguez-Vieitez et al., 2016
	61.9 ± 6.4 years			
	AD:			
	63.0 ± 6.5 years			
8AD; 8MCI	MCI:	TSPO-[11C] (R)PK11195; [11C]-PIB	Increase in MCI and AD; longitudinal reduction in MCI; longitudinal increase in AD	Fan et al., 2017
	67.7 ± 6.6 years			


*AD, Alzheimer’s disease; MCI, mild cognitive impairment; PET, positron emission tomography; TSPO, 18-kDa translocator protein; [11C]-DED, [11C]-deuterium-L-deprenyl; [18F]-DPA-714, [18F]-dimethylpyrazolo pyrimidine acetamide; [11C]-PIB, [11C]-Pittsburgh compound B; [11C]-PBR28, [11C]-peripheral benzodiazepine receptor 28; y, years; m, months.*

It should be noted that none of the current TSPO ligands are specific tracers of M1 or M2 microglia. This may be because no clear target of specific subtypes of activated microglia has been discovered to develop PET tracers (Vivash and O’Brien, 2016). Recently, some subtypes of purinergic receptors have been increasingly considered as specific markers of the M1 or M2 status of microglial cells in preclinical research. For example, PET tracers labeled with [11C] were demonstrated to have a highly specific affinity with purinergic receptor subtype 7 (P2X7R, a molecular target for pro-inflammatory microglia) in an animal model (Ory et al., 2016; Territo et al., 2017). Furthermore, a study by Beaino et al. (2017) has demonstrated that P2X7R is associated with a pro-inflammatory phenotype of human microglia, and P2Y12R is associated with an anti-inflammatory phenotype in postmortem tissues of multiple sclerosis patients, suggesting P2Y12R and P2X7R are promising targets for discriminating microglial phenotypes *in vivo* by PET imaging. Controversially, P2Y12R is considered a marker of homeostatic microglia that is downregulated in neurodegenerative disease status (Keren-Shaul et al., 2017; Zrzavy et al., 2017). Importantly, P2X7R and P2Y12R have been investigated in only animals and postmortem tissues, and their feasibility and practicability in AD patients are still unknown.

Nonetheless, we have gained many useful insights from the previous clinical studies. Most clinical studies have shown increased TSPO ligand binding in both AD patients and patients with MCI (Cagnin et al., 2001; Okello et al., 2009; Yasuno et al., 2012; Kreisl et al., 2013; Hamelin et al., 2016), indicating high microglial activation during the two conditions. MCI is defined as a transitional stage between normal function and dementia, which is not severe enough for a diagnosis of AD. Thus, *in vivo* detection of microglial activation by PET might be quite helpful in the early assessment of neuroinflammation for MCI patients. Moreover, a study by Fan et al. (2017) showed a longitudinal reduction of TSPO binding after the first peak of microglial activation in MCI patients and a longitudinal increase after the second peak of microglial activation in AD patients. The outcome of this study might be illustrated in **Figure 1**, which elucidates the discrepancies among the studies in **Table 1**. These differences, such as the age-dependent increase of TSPO ligand binding in one study (Fan et al., 2015), and its decline with age in another (Rodriguez-Vieitez et al., 2016), might stem from examination at different time points. The transition from M2 to M1 microglia in the progression of AD has also been identified in PET clinical studies; for example, the microglial activation detected by PET seems to play a protective role at the preclinical stage in AD patients (Hamelin et al., 2016; Fan et al., 2017). Combined with neurological examinations, including Mini-Mental State Examination scores, detecting microglial activation *in vivo* could indirectly indicate the current phenotype status based on the specific stage, as the M2 phenotype peaks in the MCI stage and the M1 phenotype dominates in the symptomatic AD stage. In short, by imaging microglial activation, it may be possible to indirectly identify the phenotype of activated microglia to help implement more specific and effective therapeutic approaches.

## Microglia-Targeted Therapies in AD Model Mice

Recently, an increasing number of anti-AD therapies targeting microglia have been discovered in AD animal models. Here, we categorize these new drugs or therapies into three types based on their effects on microglia: M2 enhancement, M1 suppression, and M1/M2 switching. For example, a recent study has shown that deferoxamine treatment induces M2 activation of microglia and significantly ameliorates Aβ deposition in the hippocampus of 12-month-old *APP/PS1* mice (Zhang and He, 2017). In this study, deferoxamine was also found to inhibit M1 activation of microglia and apoptosis in the brain. Thus, it is conceivable that enhancement of the M2 phenotype may be beneficial for AD treatment in the early stage.

Anti-inflammatory strategies have been proposed because the neuroinflammatory response is considered as a key factor in the pathogenesis of AD. However, it needs to be noted that anti-inflammatory strategies should mainly be targeted at the detrimental M1 phenotype rather than the neuroprotective M2 phenotype. For example, minocycline has recently been shown to inhibit the pro-inflammatory phenotype of microglia and enhance phagocytosis of Aβ deposits (El-Shimy et al., 2015). Besides M1 suppression and M2 enhancement, shifting the M1 phenotype to the M2 phenotype has been the most proposed as a potential therapeutic direction for AD treatment. An increasing number of drugs have been identified that modulate the transition of M1 to M2 in AD model mice, including Lipoxin A4 (Medeiros et al., 2013), IL-33 (Fu et al., 2016), GW2580 (Olmos-Alonso et al., 2016), EGb761 (Wan et al., 2016), and Iso-α-acids (Ano et al., 2017). This kind of regulation could generate multiple beneficial effects. They reduce the level of pro-inflammatory cytokines and increase the level of anti-inflammatory factors; additionally, microglia of the M2 phenotype have been demonstrated to have improved phagocytic function and reduce Aβ deposits. All of these effects correlate with an improved performance in cognition and memory, implying that shifting the microglial M1 phenotype to an M2 phenotype is a promising approach for treatment of AD patients.

Beyond the above drug explorations, in recent years, stem cell transplantation has been shown to effectively prevent memory impairment and improve cognitive function in AD model mice (Lee et al., 2010, 2016; Kanamaru et al., 2015). The mechanisms involved in stem cell therapies are diverse and mainly include neuronal replacement (Abdel-Salam, 2011), neurotrophic support (Marsh and Blurton-Jones, 2017), and immunomodulation (Shen et al., 2017). Immunomodulation, particularly the effect on microglia, plays a key role during stem cell transplantation for AD (Shen et al., 2017). For example, adipose-derived mesenchymal stem cell therapy can induce activated microglia into an alternatively activated phenotype in AD model mice (Ma et al., 2013). Another study has found that neural stem cell treatment in APP/PS1 mice significantly improves cognitive deficits via suppression of microglial activation (Zhang et al., 2016). In addition, bone marrow-derived mesenchymal stem cells promote microglial activation and reduce the level of Aβ deposits in the brain of an acutely induced AD model (Lee et al., 2009). These results demonstrate that stem cell therapies cannot only enhance M2 microglia but also suppress M1 microglia and shift the M1 phenotype to M2, indicating that stem cell transplantation may have a beneficial effect regardless of the treatment time point. However, when to implement this kind of therapy for the most beneficial effect is still an open question that requires further study.

Microglia-targeted therapies in AD also include genetically-defined therapeutic targets (Wes et al., 2016), treatments to restore the homeostatic microglial phenotype (Ofengeim et al., 2017) and treatments that increase Aβ uptake by microglia (Frenkel et al., 2013; Maezawa et al., 2018; Rangaraju et al., 2018). These approaches, though not purported to be associated with M2 enhancement, M1 suppression or the switch from M1 to M2 microglia, have attempted to modify microglial function from the disease state toward a more anti-inflammatory tone.

## Conclusion

In summary, microglial activation plays a significant role in the underlying pathology of AD. Typically, activated microglia adopt a protective M2 phenotype at the early stage and a detrimental M1 phenotype at the later stage of AD progression. Current imaging methodologies, such as MRI and PET, are able to detect microglial activation *in vivo*. Although there are currently no specific biomarkers for M1 or M2 microglia in clinical studies, increasing evidence suggests PET may be a useful tool to assess the extent of microglial activation in AD patients. Thus, together with behavioral function scoring, imaging of microglial activation may help clinicians indirectly identify the current microglial phenotype. After clarification of the phenotype of activated microglia, a directed therapy targeting that specific microglial phenotype should then be implemented, such as M1 suppression, M2 enhancement or M1/M2 switching. Further work is needed to develop feasible innovative PET tracers that can accurately recognize the specific phenotype of activated microglia in AD patients. Some subtypes of purinergic receptors, such as P2X7R and P2Y12R, have been validated as promising PET imaging targets for microglia phenotypes in preclinical studies. These studies will be extremely important for microglia-targeted AD therapies, given that microglial phenotypes vary according to disease stage.

## Author Contributions

RW and XB co-designed the manuscript. ZS wrote and edited the content. XB revised the draft. All authors read and approved the final manuscript.

## Conflict of Interest Statement

The authors declare that the research was conducted in the absence of any commercial or financial relationships that could be construed as a potential conflict of interest. The reviewer JV and handling Editor declared their shared affiliation at the time of the review.
